# Upcycled Animal Protein Hydrolysates for Gilthead Seabream: Impact on Growth Performance, Nutrient Utilisation, Intestinal Structure and Skeletal Muscle Gene Expression

**DOI:** 10.1155/anu/2611411

**Published:** 2026-07-17

**Authors:** A. Alves-de-Oliveira, M. Monteiro, T. Sá, A. Filipa-Silva, A. Almeida, F. Kokou, L. M. P. Valente

**Affiliations:** ^1^ CIIMAR/CIMAR LA, Interdisciplinary Centre of Marine and Environmental Research, University of Porto, Av. General Norton de Matos S/ N, Matosinhos 4450–208, Portugal, up.pt; ^2^ ICBAS, School of Medicine and Biomedical Sciences, University of Porto, Rua de Jorge Viterbo Ferreira 228, Porto 4050–313, Portugal, up.pt; ^3^ ETSA, I.T.S., Industry of By-Products, S.A. (ETSA Group), Loures 2660–119, Portugal; ^4^ Aquaculture and Fisheries Group, Wageningen University and Research, Wageningen, Netherlands, wur.nl

## Abstract

The development of local circular feed systems has increased interest in protein hydrolysates (PHs) derived from processed animal proteins (PAPs), which can upcycle protein‐rich streams into functional aquafeed ingredients. This study evaluated novel PHs sourced from insect meal (*Hermetia illucens*), fish by‐products and swine by‐products as replacements for a high‐quality fish PH (CPSP90) in diets for gilthead seabream (*Sparus aurata*). A control diet, containing 20% fish meal, 22% plant proteins, 23% PAPs and 3% CPSP90, was compared with three experimental diets in which CPSP90 was replaced by 3% of each alternative PH, resulting in the INSECT, FISH and SWINE diets. Seabream juveniles (11 g) were reared in triplicate tanks and fed the experimental diets three times per day over 88 days. A parallel digestibility trial was also conducted. All diets were well accepted across treatments (voluntary feed intake [VFI] = 1.7–1.8 g 100 g^−1^ average body weight day^−1^). Nutrient digestibility, fish final body weight (FBW; 60–65 g), feed conversion ratio (FCR; 1.1–1.2) and anterior intestine histomorphology remained similar across treatments. Fish fed the SWINE diet showed upregulation of *mrf4*, *mlc2a* and *mlc2b*, suggesting modulation of myogenic differentiation and muscle structural pathways, although this was not reflected in improved growth. Fish fed the INSECT diet showed reduced faecal lipid losses, together with downregulation of *capn3*, which may reflect metabolic adjustments related to lipid utilisation. Fish fed the FISH diet exhibited muscle gene expression patterns and growth performance similar to those of the control group. Alternative PHs showed potential to replace CPSP90 in gilthead seabream diets, supporting circular aquafeed formulations while sustaining feed efficiency and fish growth.

## 1. Introduction

With growing concerns over sustainability and the limited availability of fishmeal (FM), widely used as a protein source for carnivorous fish, its strategic use in aquafeeds has become imperative [1]. Consequently, aquafeeds have increasingly incorporated higher levels of plant proteins and processed animal proteins (PAPs), the latter mainly produced from animal by‐products (ABPs) [2, 3]. However, these alternatives are often associated with problems related to palatability, amino acid (AA) imbalance and suboptimal growth performance in farmed fish species [2, 4].

To overcome these limitations, functional diets have been explored as a promising strategy, going beyond basic nutrition by enhancing fish physiological welfare and growth performance [5]. In this context, protein hydrolysates (PHs) are increasingly recognised as valuable feed ingredients given their richness in essential AAs and bioactive peptides, which not only serve as nutrients but also exert biological functions with physiological benefits [6, 7]. Through the hydrolysis of native proteins, long protein chains are broken down into smaller peptide chains and free AAs, forming PHs [8]. From a physiological perspective, PHs should be more bioavailable than intact proteins since AAs from small peptides are more easily absorbed, having the potential to promote fish growth and development [9, 10]. Beyond their role in improving digestibility and nutrient absorption [11], the inclusion of PHs in aquafeeds may promote muscle growth by modulating genes associated with hyperplastic and hypertrophic muscle development in fish [12, 13]. These combined characteristics position PHs as valuable ingredients in modern aquafeed formulations.

Several commercially available PHs, mostly derived from marine by‐products, are already used in the aquafeed industry, such as CPSP [14], Aquabite [15], ActiFish [16] and Actipal [17]. These products are valued for their high palatability, feed attractiveness, bioavailability and potential to enhance growth performance and immune responses [11, 18]. As aquafeed production is projected to double by 2050, exceeding 100 million tonnes [3], the demand for PHs is expected to increase. This highlights the need to expand production capacity and diversify raw material sources. While marine‐derived PHs currently dominate the market, broadening the range of raw materials for PH production represents a promising direction for sustainable aquafeed formulation. Accordingly, the use of non‐marine sources is projected to increase, driven by sustainability concerns, rising competition for FM and its escalating cost [19].

In this context, PHs sourced from PAPs, including those derived from insect meal and ABPs, present a relevant solution, aligning with the European Union Zero Waste Strategy, to promote local circular feeds that recover nutrients without competing with the human food supply [3, 20]. Insects, such as black soldier fly (*Hermetia illucens*) larvae, are able to utilise a wide range of vegetable and organic by‐products, effectively converting low‐value biomass into high‐quality protein [21]. Annually, the European rendering industry processes 18 million tonnes of ABPs [22]. A subset of these materials, categorised as category 3 by‐products under European Regulation (EU) No 142/ 2011, is primarily used for animal feed [23]. Approximately 213 thousand tonnes of PAPs are incorporated in European aquafeeds annually [22], highlighting their relevance to the sector. Considering the high industrial availability of these PAPs, their transformation into PHs enables the upcycling of low‐value materials while producing an ingredient enriched in bioactive peptides and enhanced functional properties [24, 25]. Within a circular bioeconomy framework, this approach reduces organic waste while supporting sustainable aquafeed production, contributing to the United Nations Sustainable Development Goal 12 (Responsible Consumption and Production, SDG 12) [18].

To support sustainable aquafeed growth, it is crucial to explore novel PH sources and assess their nutritional and functional properties. Producing PHs from locally sourced ABPs as well as insect meal upcycles protein‐rich streams into high‐value functional ingredients, reduces reliance on imported raw materials and aligns with circular‐bioeconomy principles. This study hypothesises that PHs derived from different segments of the local agri‐food sector can sustain fish growth and overall digestibility when incorporated into diets for gilthead seabream (*Sparus aurata*), a widely farmed species across Europe and the Mediterranean. To test this hypothesis, three PHs, one from insect larvae meal, one from fishery by‐products and one from swine by‐products, were incorporated into diets for seabream, and their effects on apparent digestibility, nutrient utilisation, growth responses, feed efficiency, intestinal morphology and gene expression in skeletal muscle were evaluated.

## 2. Materials and Methods

### 2.1. Ethical Statement

Experimental procedures were authorised by the CIIMAR Ethics Committee (ORBEA‐CIIMAR 37‐2023) and performed in accordance with EU Directive 2010/63, Portuguese legislation (Decreto‐lei 113/ 2013), and FELASA Category C guidelines under accredited scientific supervision.

### 2.2. Experimental Hydrolysates

Three PHs were tested: an insect hydrolysate (IH), a fish hydrolysate (FH) and a swine hydrolysate (SH). IH was obtained from *Hermetia illucens* larvae meal, acquired from the Hermetia Baruth GmbH, Germany, whereas FH was produced from fish by‐products supplied by the Portuguese canning and fish processing industries. Both IH and FH were obtained by alcalase enzymatic hydrolysis. SH was obtained from monospecies swine Category 3 by‐products collected from slaughterhouses in Portugal and processed according to Regulation (EU) No 142/ 2011 and subjected to physical hydrolysis using pressure and heat, without chemical addition (Patent Application PCT/ IB2024/061806). The PHs were produced by ETSA‐SGPS, S. A. (Empresa Transformadora de Subprodutos Animais, Lisbon, Portugal), as described by Monteiro et al. [26]. Following hydrolysis, the protein fraction was concentrated with a rotary evaporator and spray‐dried prior to incorporation into the experimental diets (Table 1). Detailed characterisation of the hydrolysates is available in Monteiro et al. [26]. IH and FH were predominantly composed of low‐to medium‐MW peptides (1–3 kDa), followed by small‐MW peptides (MW <1 kDa). Conversely, CPSP90 and SH displayed broader MW distributions, with SH mainly composed of large MW peptides (MW >5 kDa).

**Table 1:** Ingredients of the experimental diets.

Ingredient (%)	CTRL	INSECT	FISH	SWINE
Fishmeal super prime^a^	13.0	13.0	13.0	13.0
Fishmeal 60^b^	7.0	7.0	7.0	7.0
Fish protein hydrolysate^c^	3.0	—	—	—
Insect hydrolysate (IH)^d^	—	3.0	—	—
Fish hydrolysate (FH)^e^	—	—	3.0	—
Swine hydrolysate (SH)^f^	—	—	—	3.0
Poultry meal^g^	15.0	15.0	15.0	15.0
Poultry blood meal^h^	3.0	3.0	3.0	3.0
Feather meal hydrolysate^i^	5.0	5.0	5.0	5.0
Microbial protein biomass^j^	4.0	4.0	4.0	4.0
Corn gluten meal^k^	8.0	8.0	8.0	8.0
Guar meal^l^	5.3	5.3	5.3	5.3
Rapeseed meal^m^	3.5	3.5	3.5	3.5
Wheat meal^n^	11.3	11.1	11.0	11.0
Whole peas^o^	5.5	5.5	5.5	5.5
Vitamin and mineral premix^p^	1.0	1.0	1.0	1.0
Choline chloride 50^q^	0.2	0.2	0.2	0.2
Antioxidant^r^	0.2	0.2	0.2	0.2
Sodium propionate^s^	0.1	0.1	0.1	0.1
Monoammonium phosphate^t^	0.3	0.3	0.3	0.3
Rapeseed lecithin^u^	0.5	0.5	0.5	0.5
Fish oil^v^	6.8	6.8	6.8	6.8
Rapeseed oil^w^	7.3	7.5	7.6	7.6

*Note:* Commercial name: CP%, CF%, Supplier, Country.

^a^Diamante (66.3% crude protein [CP] and 11.5% crude fat [CF]) Pesquera Diamante, Peru.

^b^CONRESA (61.2% CP and 8.4% CF), Conserveros Reunidos S.A., Spain.

^c^CPSP90 (83.8% CP and 8.4% CF), Sopropêche, France.

^d^Hydrolysate from insect meal: enzymatic hydrolysis with alcalase (55.1% CP and 2.04% CF), ETSA, Portugal.

^e^Hydrolysate from fish by‐products (multi‐species): enzymatic hydrolysis with alcalase (80.5% CP and 0.10% CF), ETSA, Portugal.

^f^Hydrolysate from pig by‐products (mono‐species): pressure and heat hydrolysis (87.5% CP and 0.20% CF), ETSA, Portugal.

^g^Poultry meal (62.4% CP and 12.5% CF) SAVINOR UTS, Portugal.

^h^Poultry blood meal (89% CP and 0.47% CF), SONAC, The Netherlands.

^i^Feather meal hydrolysate (88.8% CP and 1.6% CF), Empro Europe NV, The Netherlands.

^j^Aminopro NT70 (74.1% CP and 3.1% CF) (*Corynebacterium glutamicum*), MAZZOLENI SPA, Italy.

^k^Corn gluten meal (61.2% CP and 5.2% CF) COPAM, Portugal.

^l^Guar Korma (55.3% CP and 7.8% CF) KFEED Ltd., Bulgaria.

^m^Rapeseed meal (34.3% CP and 2.1% CF) solvent extracted, Ribeiro e Sousa Lda, Portugal.

^n^Wheat meal (11.7% CP and 1.6% CF), Molisur, Spain.

^o^Whole peas (19.8% CP and 1.1% CF), PREMIX Lda. Portugal.

^p^Vitamins (IU or mg kg^−1^ diet): DL‐alphatocopherol acetate, 100 mg; sodium menadione bisulphate, 25 mg; retinyl acetate, 20,000 IU; DL‐cholecalciferol, 2000 IU; thiamine, 30 mg; riboflavin, 30 mg; pyridoxine, 20 mg; cyanocobalamin, 0.1 mg; nicotidin acid, 200 mg; folic acid, 15 mg; ascorbic acid, 1000 mg; inositol, 500 mg; biotin, 3 mg; calcium pantothenate, 100 mg; choline chloride, 1000 mg, betaine, 500 mg. Minerals (g or mg kg^−1^ diet): cobalt carbonate, 0.65 mg; copper sulphate, 9 mg; ferric sulphate, 6 mg; potassium iodide, 0.5 mg; manganese oxide, 9.6 mg; sodium selenite, 0.01 mg; zinc sulphate. 7.5 mg; sodium chloride, 400 mg; calcium carbonate, 1.86 g; excipient wheat middling’s. Premix Lda, Portugal.

^q^Choline chloride 50, ORFFA, The Netherlands.

^r^VERDILOX, Kemin Europe NV, Belgium.

^s^Sodium propionate, Disproquímica, Portugal.

^t^Monoammonium phosphate (26% P), Phosphea, Serbia.

^u^CANOLECITHIN F60 (94% CF), Novastell, France.

^v^Fish oil (98.1% CF; 16% EPA; 12% DHA), Sopropêche, France.

^w^Rapeseed oil (98.2% CF) JC Coimbra, Portugal.

### 2.3. Experimental Diets

A commercial‐style control diet (CTRL) was formulated with a blend of protein sources, including plant proteins (22%) and PAPs (23%), limiting FM inclusion to 20% (13% FM Super Prime + 7% FM 60). The CTRL diet contained 3% CPSP90, a high‐quality commercial fish PH, reflecting current industry formulations and the practical inclusion level of PHs in aquafeeds. Three additional experimental diets ( INSECT, FISH and SWINE) were obtained by replacing CPSP90 with 3% of each PH (IH, FH and SH). Chromium oxide (Cr_2_O_3_) was incorporated into each diet at 1% as an inert marker for the determination of apparent digestibility coefficients (ADCs). Diets were formulated based on gilthead seabream nutritional requirements [27], extruded (2 mm) by SPAROS, Lda (Olhão, Portugal) and stored at 4°C. Diet composition is detailed in Tables 1 and 2, and fatty acid profile in Table S1.

**Table 2:** Proximate composition and amino acid (AA) profile of the experimental diets.

Parameter	CTRL	INSECT	FISH	SWINE
Proximal composition (g 100 g^−1^ DM or kJ g^−1^ DM)				
Dry matter	95.93	97.53	96.58	97.57
Crude protein	49.99	50.94	51.61	52.88
Crude fat	19.85	20.18	20.03	19.88
Ash	7.87	9.71	9.26	9.59
Energy	22.65	22.83	22.86	22.97
Phosphorus	0.88	0.98	1.03	1.04
Digestible protein (DP)	40.13	40.98	39.89	41.88
Digestible energy (DE)	18.59	18.95	18.74	19.18
DP:DE (mg kJ ^−1^)	21.59	21.62	21.28	21.83
AA profile (g 100 g^−1^ DM)				
Essential amino acids (EAAs)				
Arginine	2.95	2.80	2.91	2.91
Histidine	1.06	1.10	1.11	1.10
Lysine	2.55	2.68	2.76	2.69
Threonine	2.30	2.25	2.33	2.27
Isoleucine	2.16	2.20	2.27	2.23
Leucine	3.75	3.69	3.74	3.93
Valine	2.34	2.47	2.50	2.48
Methionine	1.15	1.13	1.24	1.16
Phenylalanine	2.16	2.18	2.23	2.22
Σ EAA	20.42	20.50	21.08	21.00
Non‐essential amino acids (NEAAs)				
Cystine	1.06	1.04	1.06	1.09
Tyrosine	1.92	1.86	1.99	1.90
Aspartic acid + asparagine	3.75	3.76	3.83	3.78
Glutamic acid + glutamine	7.19	6.97	7.26	7.30
Alanine	2.58	2.72	2.82	2.90
Glycine	2.61	2.77	2.91	3.08
Proline	2.65	2.74	2.82	3.09
Serine	2.31	2.42	2.52	2.57
Taurine	0.18	0.17	0.19	0.17
Σ NEAA	24.26	24.43	25.40	25.88
EAA/NEAA	0.84	0.84	0.83	0.81
ƩAA	44.68	44.92	46.48	46.88

*Note*: The values of DP, DE and DP:DE were calculated using the mean value of apparent digestibility coefficients (ADCs) for crude protein and gross energy reported in Table 3. Taurine was included in the sum of NEAA.

Abbreviations: AA, amino acid; DE, digestible energy; DM, dry matter; DP, digestible protein; EAA, essential amino acids; NEAA, non‐essential amino acids.

**Table 3:** Apparent digestibility coefficients (ADC, %) of the nutrients and energy in the experimental diets and nutrient balances of *Sparus aurata* fed the experimental diets for 88 days.

Parameter	CTRL	INSECT	FISH	SWINE	*p*‐Value
ADC (%)					
Dry matter	62.21 ± 4.55	61.74 ± 5.25	56.97 ± 3.97	59.72 ± 3.71	0.493
Crude protein	80.27 ± 3.04	80.45 ± 3.29	77.29 ± 2.96	79.19 ± 2.91	0.590
Crude fat	92.49 ± 0.93	93.39 ± 0.92	92.63 ± 0.14	93.86 ± 0.31	0.114
Gross energy	82.07 ± 2.72	83.01 ± 4.87	81.99 ± 2.64	83.52 ± 1.44	0.916
Phosphorus (Phos)	61.56 ± 7.95	61.52 ± 5.60	60.27 ± 5.81	61.53 ± 10.00	0.996
Cobalt (Co)	51.40 ± 7.90	50.47 ± 11.12	46.90 ± 1.12	55.26 ± 0.78	0.550
Copper (Cu)	43.91 ± 8.29	43.74 ± 13.15	48.14 ± 1.53	47.14 ± 2.06	0.864
Manganese (Mn)	36.11 ± 13.46	37.95 ± 14.72	38.35 ± 4.03	44.25 ± 5.72	0.801
Nitrogen (N) balance (g kg ABW^−1^ day^−1^)					
Digestible N intake (DN)	1.165 ± 0.009	1.123 ± 0.020	1.106 ± 0.057	1.195 ± 0.038	0.060
N gain	0.469 ± 0.000	0.475 ± 0.004	0.486 ± 0.013	0.487 ± 0.011	0.093
N retention efficiency (NRE, %DN)	40.24 ± 0.32	42.33 ± 1.11	44.08 ± 3.08	40.77 ± 1.68	0.122
Faecal N losses	0.286 ± 0.002^b^	0.273 ± 0.005^b^	0.325 ± 0.017^a^	0.314 ± 0.010^a^	0.001
Metabolic N losses	0.696 ± 0.009	0.648 ± 0.024	0.619 ± 0.064	0.708 ± 0.041	0.083
Lipid (L) balance (g kg ABW^−1^ day^−1^)					
Digestible L intake (DL)	3.33 ± 0.03	3.23 ± 0.06	3.22 ± 0.17	3.33 ± 0.11	0.443
L gain	2.38 ± 0.19	2.47 ± 0.09	2.32 ± 0.02	2.47 ± 0.04	0.320
L retention efficiency (LRE, %DL)	71.34 ± 5.59	76.52 ± 4.34	72.40 ± 3.22	74.35 ± 3.43	0.497
Faecal L losses	0.270 ± 0.002^a^	0.229 ± 0.004^b^	0.256 ± 0.013^a^	0.218 ± 0.007^b^	0.001
Metabolic L losses	0.953 ± 0.184	0.760 ± 0.152	0.891 ± 0.148	0.857 ± 0.143	0.535
Energy (E) balance (kJ kg ABW^−1^ day^−1^)					
Digestible E intake (DE)	337.05 ± 2.62	324.72 ± 5.77	324.77 ± 16.80	342.40 ± 10.84	0.170
E gain	148.80 ± 5.76	149.42 ± 2.68	150.26 ± 1.56	157.01 ± 3.81	0.093
E retention efficiency (ERE, %DE)	44.15 ± 1.82	46.03 ± 1.59	46.33 ± 1.97	45.91 ± 2.49	0.562
Metabolisable E	319.73 ± 2.40	308.59 ± 5.18	309.34 ± 15.24	324.75 ± 9.87	0.180
Faecal E losses	73.65 ± 0.57^a^	66.47 ± 1.18^b^	71.33 ± 3.69^ab^	67.54 ± 2.14^b^	0.014
Branchial + urinary E losses	17.33 ± 0.23	16.14 ± 0.59	15.43 ± 1.59	17.64 ± 1.01	0.083
Total heat production	170.92 ± 6.78	159.17 ± 7.57	159.09 ± 13.71	167.74 ± 13.54	0.474
Phosphorus (Phos) balance (g kg ABW^−1^ day^−1^)					
Digestible Phos intake (DPhos)	0.099 ± 0.001^c^	0.103 ± 0.002^bc^	0.108 ± 0.006^ab^	0.114 ± 0.003^a^	0.003
Phos gain	0.065 ± 0.008	0.064 ± 0.014	0.066 ± 0.011	0.077 ± 0.020	0.617
Phos retention efficiency (PhosRE, %DPhos)	65.96 ± 8.17	61.80 ± 13.16	61.16 ± 11.01	67.69 ± 17.53	0.906
Faecal Phos losses	0.062 ± 0.001^b^	0.064 ± 0.001^b^	0.071 ± 0.004^a^	0.071 ± 0.002^a^	0.001
Metabolic Phos losses	0.033 ± 0.008	0.039 ± 0.013	0.042 ± 0.014	0.037 ± 0.020	0.895

*Note:* Values represent mean ± SD (*n* = 3). In each row, different superscript lowercase letters indicate significant differences between treatments (*p* < 0.05).

Abbreviations: ABW, average body weight; ADC, apparent digestibility coefficients; DE, digestible energy; DL, digestible lipids; DN, digestible nitrogen; DPhos, digestible phosphorus; E, energy; ERE, energy retention efficiency; L, lipids; LRE, lipid retention efficiency; N, nitrogen; NRE, nitrogen retention efficiency; Phos, phosphorus; PhosRE, phosphorus retention efficiency.

### 2.4. Experimental Trials

#### 2.4.1. Growth Trial and Sampling

The growth trial was conducted at CIIMAR using juvenile gilthead seabream (*Sparus aurata*), acquired from a commercial hatchery (Grupo Culmarex, Murcia, Spain). Fish were quarantined for 15 days to acclimate to the new conditions and were hand‐fed twice daily with a commercial diet at 1.5% of body weight (Aquasoja, Sorgal S.A.; 50% crude protein, 20% crude fat, dry matter (DM) basis). After acclimation, fish were fasted for 24 h, individually weighed (11.1 ± 1.1 g) and measured for total length (9.4 ± 0.4 cm) under light anaesthesia (60 μL L^−1^ of 2‐phenoxyethanol; Sigma–Aldrich, Massachusetts, USA). Then, fish were distributed into 12 experimental groups with similar average weights and stocked in 160‐L fibreglass tanks within a recirculating aquaculture system (RAS; 65 fish tank^−1^, variation coefficient of 9.8% within tanks; stocking density of 4.5 kg m^−3^). Abiotic conditions were maintained at 20 ± 1°C, salinity 35 ± 1‰, filtered seawater flow rate of 10 L min^−1^, and a 12 h light/12 h dark photoperiod. For initial whole‐body composition (WBC), 20 fish from the starting population were euthanised by anaesthetic overdose (500 μL L^−1^), pooled and stored at −20°C until analysis.

Each diet was randomly assigned to three replicate tanks. Fish were fed to apparent satiation three times daily (9:00, 12:00 and 16:00) for 88 days using automatic feeders. The feed amount was adjusted daily according to consumption: increased by 5% when all pellets were consumed and reduced by 5% when uneaten pellets remained. Uneaten pellets were siphoned, oven‐dried and weighed to calculate actual feed intake. Water parameters were regularly monitored and maintained within optimal levels for marine species [28]. At the end of the growth trial, fish were fasted for 24 h and lightly anesthetised, as previously described. All fish were individually weighed and measured to assess growth performance parameters and feed consumption was recorded. Five fish per tank (*n* = 15 per treatment) were euthanised by spinal cord section under anaesthesia to be sampled. Both viscera and the liver were weighed to calculate somatic indices. A 1 cm portion of the anterior intestine, 0.5 cm after the pyloric caeca, was sampled, rinsed and fixed in 4% formaldehyde (pH 7.0). A 25 mg skinless fast‐twitch skeletal muscle sample was collected from the dorsal left region, after the fish head, frozen in liquid nitrogen and stored at −80°C for subsequent gene expression analyses. An additional five fish per tank were euthanised and stored at −20°C to determine the final WBC.

#### 2.4.2. Digestibility Trial

In parallel with the growth trial, a digestibility trial was conducted in a RAS Guelph system with 12 fibreglass tanks of 50 L for faeces collection, each equipped with an individual faeces sedimentation column connected to the outlet of each tank [29]. Twelve homogeneous groups of 17 fish (body weight: 11.1 ± 2.4 g) were randomly allocated to these tanks (density of 3.8 kg m^−3^), following the same randomisation procedure used in the growth trial, with each diet assigned to three replicate tanks. Abiotic conditions were identical to those of the growth trial. Before faeces collection, fish were fed the experimental diets for a 30‐day adaptation period. During this period, automatic feeders delivered feed three times daily, and fish were fed until no further consumption was observed. Thereafter, feed distribution was standardised across tanks using the lowest average intake tank to ensure uniform feed intake during the faecal collection. To prevent contamination, tanks and settling columns were thoroughly cleaned daily after the final meal. Sedimentation columns were kept on ice overnight to limit faecal microbial degradation [30]. Faeces were collected every morning before the first meal, centrifuged for 10 min at 3000 × *g* at 4°C and stored daily at −20°C. Faeces were collected for 43 days. Faecal samples from each tank were then pooled, freeze‐dried, finely ground and sieved to obtain a homogeneous material for proximate composition analysis.

### 2.5. Chemical Analyses

#### 2.5.1. Proximate Composition of Experimental Diets, Whole‐Fish and Faecal Samples

Prior to whole‐body composition analysis, five fish per tank were pooled into one sample (*n* = 3 per treatment) *and* freeze‐dried. Proximate composition of diets, whole‐fish and faeces was determined in duplicate following AOAC [31] methods. Moisture was determined by oven‐drying samples at 105°C for 24 h, and ash by incineration at 550°C for 5 h. Gross energy values were obtained by a bomb calorimeter (IKA C2000, IKA‐Werke GmbH & Co.KG, Staufen, Germany). Nitrogen (N) was analysed by Dumas combustion using a Leco FP‐528 nitrogen analyser (Leco Corporation, St. Joseph, USA) and converted to crude protein using a factor of 6.25. Crude fat in diets and whole‐fish was extracted with petroleum ether using a Soxtec 2055 apparatus (Foss, Höganäs, Sweden) and faecal lipids were determined according to Folch et al. [32]. Following ash digestion in HCl at 150°C, total phosphorus (Phos) was quantified as phosphate using ammonium molybdate, with the absorbance read at 820 nm [33].

#### 2.5.2. Chromic Oxide Content and Mineral Analysis

The chromic oxide concentration in diets and faeces was measured using the method of Bolin et al. [34], in triplicate. Other minerals, including aluminium (Al), calcium (Ca), cobalt (Co), copper (Cu), iron (Fe) and manganese (Mn), were analysed in diets and faeces following the method described by Azevedo et al. [35]. Dry samples (400 mg) were subjected to microwave digestion with HNO_3_ and H_2_O_2_ in Teflon vessels (Milestone MLS 1200 M, Italy). The diluted digests were analysed for mineral content by inductively coupled plasma mass spectrometry (iCAP Q, Thermo Fisher Scientific, Germany).

#### 2.5.3. AA and Fatty Acid Composition

AA composition of diet samples was determined after 48 h HCl hydrolysis by ultra‐high‐performance liquid chromatography, as described by Teodósio et al. [36]. AA levels were expressed as g 100 g^−1^ DM, and the sum of non‐essential AAs (NEAAs) includes taurine. Dietary fatty acids were derivatised by direct acid transmethylation as described in Parrish et al. [37], with modifications described in Monteiro et al. [38]. Fatty acid methyl esters (FAMEs) were identified and quantified by gas chromatography (Shimadzu Nexis GC‐2030, Kyoto, Japan), using tricosanoic acid (C23:0) as the internal standard. Fatty acid composition was reported in g 100 g^−1^ DM.

### 2.6. Anterior Intestine Histology

After fixation in 4% formaldehyde, anterior intestine samples were preserved in 70% ethanol, embedded in paraffin, sectioned at 4 μm using a semi‐automated rotary microtome (Leica RM 2245, Leica Biosystems, Nussloch, Germany), stained with alcian blue/periodic acid schiff (AB/ PAS; pH 2.5) and scanned at 20x (Pannoramic 1000 Flash DX, 3DHistech, Budapest, Hungary). Three fish per tank, representative of the average final body weight (FBW) of the tank, were selected for histological evaluation (*n* = 9), with one cross‐section analysed per fish. Quantitative morphometric analyses were conducted as detailed by Ferreira et al. [39] using cellSens Standard 2.2 software (Olympus Corporation, Tokyo, Japan). Parameters included cross‐sectional perimeter (mm), muscularis thickness (μm), submucosa thickness (μm), villi length (μm), lamina propria width (μm), absorption area (mm^2^) and number of acid (blue) and neutral (magenta) goblet cells (GCs), as well as the area occupied by these cells. Minor adjustments were made to the protocol. Muscularis thickness was calculated from the difference between the radius of the full cross‐section and the radius of the same cross‐section measured excluding the muscularis layer. Villi length was measured from the villus apex to the muscularis layer in the eight tallest villi per section. Submucosa thickness was measured from the outer to the inner limits of the submucosa layer in the selected villi. Mean values were calculated per section.

### 2.7. Assessment of Key Skeletal Muscle Growth‐Related Gene Expression

Skeletal muscle RNA was isolated using the Direct‐zol RNA MiniPrep Kit (Zymo Research, Irvine, California, USA). Approximately 25 mg of tissue was homogenised in 800 μL TRIzol for 60 sec at 4500 rpm using a Precellys 24 Touch homogeniser (Bertin Technologies, Montigny‐Le‐Bretonneux, France), followed by RNA extraction according to the manufacturer’s protocol. Purified RNA was eluted in 25 μL DNase/RNase‐free water. RNA concentration and purity were assessed spectrophotometrically using a DeNovix DS‐11 FX instrument (DeNovix Inc., Wilmington, DE, USA) based on 260/ 280 and 260/ 230 nm absorbance ratios, and RNA was then preserved at −80°C until cDNA conversion. Complementary DNA was synthesised from 200 ng of total RNA using the NZY First‐Strand cDNA Synthesis Kit (NZYTech, Lisbon, Portugal). cDNA concentration and purity were similarly assessed before storage at −20°C until gene expression analyses.

Real‐time qPCR data were collected and analysed using a QuantStudio 3 real‐time PCR System (Thermo Fisher Scientific, Waltham, Massachusetts, USA). Primer efficiency was determined using seven serial two‐fold dilutions of a pooled cDNA sample. Efficiency was calculated from the slope of the regression line obtained from the quantification cycle (Ct) against the log_10_ of the cDNA dilution series, following the MIQE guidelines [40].

Primer sequences, annealing temperatures, qPCR amplification efficiencies, GenBank accession numbers and references are provided in Table S2. Muscle gene expression was analysed in the same fish used for histology. Target genes included reference genes (*rps18* and *rpl27a*); growth hormone/insulin‐like growth factor (*GH/IGF*) system‐related genes (*igfr-1a*, *igfr-2*, *ghr-1* and *ghr-2*); myogenic markers (*myf5*, *myod1*, *myod2*, *myog*, *mrf4*, *mymk* and *fgf6*); muscle growth regulators (*mstn*, *fst* and *murf1*); calpains (*capn1* and *capn3*); and muscle structural genes (*mhc*, *mlc2a* and *mlc2b*).

qPCR reactions were performed with 5 μL of SsoAdvanced Universal SYBR Green Supermix (Bio‐Rad Laboratories, Hercules, California, USA), 150–300 nM of each primer, and 1 μL of cDNA. Thermal cycling consisted of 98°C for 30 s, followed by 45 cycles of 95°C for 15 s and gene‐specific annealing temperatures at 55–68°C (Table S2) for 30–60 s. Specificity was confirmed by post‐amplification dissociation curves (65–95°C, 0.5°C increments). Samples and negative controls (with autoclaved H_2_O instead of cDNA) were run in technical triplicate. Relative gene expression was calculated using the comparative Ct (ΔΔCt) method [41], with *rps18* and *rpl27a* as reference genes, normalised to the CTRL diet and expressed as fold‐change.

### 2.8. Calculations

ADCs, growth performance and feed efficiency were calculated as described in Costa et al. [42]. Briefly, considering that *W*_i_ and *W*_f_ are the fish’s initial and final weights (g), respectively, and ABW is the average body weight (kg) ([*W*_f_ + *W*_i_]/2), the ADC of the experimental diets and nutrient and energy balance were calculated as follows: ADC of DM (%) = (1 − [dietary Cr level/faeces Cr level]) × 100; nutrient (or energy or mineral) ADC (%) = (1 − [dietary Cr level/faeces Cr level] × [faeces nutrient (or energy or mineral) level/dietary nutrient (or energy or mineral) level]) × 100; digestible nutrient (or energy) intake (g kg^−1^ ABW day^−1^ or kJ kg^−1^ ABW day^−1^) = nutrient (or energy) intake × ADC nutrient (or energy); nutrient (or energy) gain (g kg^−1^ ABW day^−1^ or kJ kg^−1^ ABW day^−1^) = (*W*_f_ × nutrient [or energy] content in final whole‐body − *W*_i_ × nutrient [or energy] content in initial whole‐body)/ABW/days; nutrient (or energy) retention efficiency (% digestible intake) = nutrient (or energy) gain/digestible nutrient (or energy) intake × 100; faecal nutrient (or energy) losses (g kg^−1^ ABW day^−1^ or kJ kg^−1^ ABW day^−1^) = nutrient (or energy) intake × (1 − ADC nutrient [or energy]/100); metabolic nutrient losses (g kg^−1^ ABW day^−1^) = digestible nutrient intake − nutrient gain; metabolisable energy (kJ kg^−1^ ABW day^−1^) = digestible energy (DE) intake − branchial and urinary energy losses; branchial and urinary energy losses (kJ kg^−1^ ABW day^−1^) = metabolic nitrogen losses × 24.9 kJ; total heat production (kJ kg^−1^ ABW day^−1^) = metabolisable energy − energy gain.

Formulas for growth performance and feed efficiency parameters were calculated as the following: weight gain (g) = *W*_f_ − *W*_i_; condition factor (K) = *W*_f_/(final total length)^3^ × 100; daily growth index (DGI, g day^−1^) = [(*W*_f_^1/3^ − *W*_i_^1/3^)/days] × 100; voluntary feed intake (VFI, g 100 g^−1^ ABW day^−1^) = dry feed intake/ABW/days × 100; feed conversion ratio (FCR) = dry feed intake/(*W*_f_ − *W*_i_); hepatosomatic index (HSI, %) = (liver weight/*W*_f_) × 100; viscerosomatic index (VSI, %) = (viscera weight/*W*_f_) × 100.

### 2.9. Statistical Analyses

Normality and homogeneity of variances were evaluated for all collected data using the Kolmogorov–Smirnov and Levene’s tests, respectively. Gene expression data were transformed using log (x + 1) to meet analysis of variance ( ANOVA) assumptions. Statistical differences among dietary treatments were assessed using one‐way ANOVA. In cases of significant results from the ANOVA, the Duncan test was employed for post hoc mean comparisons. A significance threshold of *p*  < 0.05 was applied. Statistical procedures were carried out using the IBM SPSS version 28.0 (IBM Corp, 2021).

## 3. Results

### 3.1. Experimental Diet Characterisation

All experimental diets were isolipidic and isoenergetic. Crude protein ranged from 50% to 53% DM and total AA ranged from 45% to 47% DM (Table 2), with the SWINE diet presenting the highest values. Diets exhibited a similar AA profile, although FISH and SWINE diets exhibited higher quantities of NEAA compared with the CTRL diet, with the SWINE diet having the highest quantities of alanine, glycine, proline and serine. The SWINE diet showed the highest digestible protein (DP) and DE, contributing to the highest DP:DE ratio observed (21.8) among experimental diets. The opposite was verified for the FISH diet (DP:DE of 21.3). Ash content varied between 7.8% and 9.7% DM. The lowest levels of saturated fatty acids (SFAs), monounsaturated fatty acids (MUFAs) and polyunsaturated fatty acids (PUFAs) were observed in the CTRL diet, whereas the SWINE diet presented the highest levels (Table S1).

### 3.2. Apparent Digestibility of the Experimental Diets and Fish Nutrient Balance

The ADCs for crude protein (77%–80%), crude fat (92%–93%), gross energy (82%–83%), Phos (60%–61%), Co (47%–55%), Cu (44%–48%) and Mn (36%–44%) showed no differences among the experimental diets (Table 3). ADC values for the remaining minerals (Al, Ca and Fe) were not determined due to high levels in the faeces that would bias calculations, likely resulting from their presence in saltwater. Regarding nutrient balances (Table 3), fish fed the FISH diet showed a trend towards the lowest digestible N intake (*p* = 0.060), and faecal N losses were significantly higher than CTRL but comparable to SWINE. Despite these differences, metabolic N losses were comparable among treatments, resulting in no significant differences in NRE (%DN). Digestible lipid intake was similar across groups. Faecal lipid losses were significantly lower in the INSECT and SWINE groups than in the other treatments. As for energy (E), the CTRL group showed significantly higher faecal E losses. Digestible Phos (DPhos) intake was statistically higher in the SWINE and FISH groups compared with CTRL. In these groups, faecal Phos losses were significantly higher compared to those of the remaining treatments. Despite this, Phos gain and PhosRE (%DPhos) did not differ among treatments.

### 3.3. Growth Performance and Whole‐Body Composition

The experimental diets were well‐accepted by gilthead seabream juveniles, as reflected by a similar VFI (1.7–1.8 g 100 g^−1^ ABW day^−1^) (Table 4). Survival was high (93%–99%) and consistent across treatments, with the few losses observed attributed to cannibalistic behaviour. After 88 days, fish from all experimental groups increased their initial body weight by 5.7‐fold, reaching an average of 62.6 g. The average FBW was similar across experimental groups. Likewise, no significant differences were observed for K, DGI and FCR. Regarding somatic indices and final WBC, no significant differences were observed (Table 4).

**Table 4:** Growth performance, feed utilisation and somatic indexes and final whole‐body composition (WBC) of *Sparus aurata* fed the experimental diets for 88 days.

Parameter	CTRL	INSECT	FISH	SWINE	*p*‐Value
Growth performance					
Final BW (g)	60.30 ± 4.12	62.71 ± 2.45	61.89 ± 1.05	65.47 ± 2.04	0.194
Weight gain (g)	49.22 ± 4.16	51.62 ± 2.45	50.83 ± 0.99	54.39 ± 2.05	0.197
K	1.62 ± 0.02	1.63 ± 0.03	1.62 ± 0.03	1.64 ± 0.03	0.809
DGI	1.92 ± 0.10	1.98 ± 0.06	1.96 ± 0.02	2.05 ± 0.05	0.188
VFI (g 100 g^−1^ ABW day^−1^)	1.81 ± 0.01	1.71 ± 0.03	1.73 ± 0.09	1.78 ± 0.06	0.161
FCR	1.16 ± 0.03	1.08 ± 0.02	1.09 ± 0.05	1.11 ± 0.03	0.082
Survival (%)	98.89 ± 0.96	93.33 ± 5.00	97.22 ± 2.55	97.78 ± 2.55	0.225
Somatic indexes (%)					
HSI	1.77 ± 0.06	1.73 ± 0.02	1.61 ± 0.03	1.64 ± 0.14	0.126
VSI	6.26 ± 0.84	6.11 ± 0.60	5.79 ± 0.61	5.61 ± 0.40	0.605
WBC (%WW)					
Moisture	67.30 ± 0.60	67.04 ± 0.09	67.30 ± 0.31	66.20 ± 1.27	0.279
Protein	16.40 ± 0.23	16.48 ± 0.14	16.88 ± 0.44	16.71 ± 0.47	0.369
Lipids	12.85 ± 0.83	13.25 ± 0.49	12.53 ± 0.07	13.17 ± 0.24	0.333
Energy (kJ g^−1^)	8.18 ± 0.22	8.15 ± 0.12	8.21 ± 0.01	8.47 ± 0.23	0.163
Ash	3.17 ± 0.36	3.19 ± 0.50	3.21 ± 0.16	3.50 ± 0.56	0.729
Phosphorus	0.44 ± 0.05	0.43 ± 0.08	0.44 ± 0.06	0.49 ± 0.11	0.745

*Note:* Initial WBC (% or kJ g^−1^ WW): moisture (%), 72.09; protein, 16.48; lipids, 7.01; energy, 6.17. Values represent mean ± SD (*n* = 3). In each row, different superscript lowercase letters indicate significant differences between treatments (*p* < 0.05).

Abbreviations: ABW, average body weight; BW, body weight; DGI, daily growth index; FCR, feed conversion ratio; HSI, hepatosomatic index; K, condition factor; VFI, voluntary feed intake; VSI, viscerosomatic index; WW, wet weight.

### 3.4. Histomorphological Analyses of the Anterior Intestine

The structure of the anterior intestine was well‐preserved in all samples (Figure S1). Morphometric parameters did not differ significantly among the dietary groups (Table 5). Total GC number tended to be lower in fish fed the diets containing the experimental hydrolysates (*p* = 0.054), given by the lowest number of acid GC in these groups (*p* = 0.060).

**Table 5:** Histomorphology of the anterior intestine of gilthead seabream juveniles fed the experimental diets for 88 days.

Parameter	CTRL	INSECT	FISH	SWINE	*p*‐Value
Cross‐sectional perimeter (mm)	11.24 ± 1.48	10.36 ± 1.94	11.47 ± 1.56	11.11 ± 1.80	0.541
Absorption area (mm^2^)	5.12 ± 1.01	4.46 ± 1.47	5.17 ± 1.18	4.84 ± 1.29	0.613
Muscularis thickness (μm)	102.58 ± 24.33	95.66 ± 15.69	97.31 ± 19.51	90.46 ± 13.34	0.582
Submucosa thickness (μm)	76.84 ± 15.22	65.08 ± 18.35	71.78 ± 14.63	70.28 ± 17.47	0.544
Lamina propria width (μm)	38.45 ± 6.24	38.59 ± 8.60	37.17 ± 5.24	34.83 ± 3.59	0.519
Villus length (μm)	1185.22 ± 171.6	1015.63 ± 195.4	1160.47 ± 162.2	1063.09 ± 192.5	0.186
Total goblet cells (GC)	3134.8 ± 723.5	2191.6 ± 400.1	2603.1 ± 394.3	2654.8 ± 856.7	0.054
Acid GC	3026.9 ± 720.2	2112.4 ± 386.4	2538.0 ± 380.1	2582.6 ± 847.8	0.060
Neutral GC	107.9 ± 44.9	79.3 ± 35.2	65.1 ± 27.8	72.2 ± 35.9	0.131
Average area acid GC (μm^2^)	85.0 ± 14.6	89.9 ± 11.8	91.8 ± 13.3	90.8 ± 10.3	0.712
Average area neutral GC (μm^2^)	33.6 ± 8.5	35.3 ± 5.2	39.9 ± 5.4	36.2 ± 6.3	0.270

*Note:* Values are the mean ± SD (*n* = 9).

### 3.5. Expression of Key Skeletal Muscle Growth‐Related Genes

**Figure 1:**
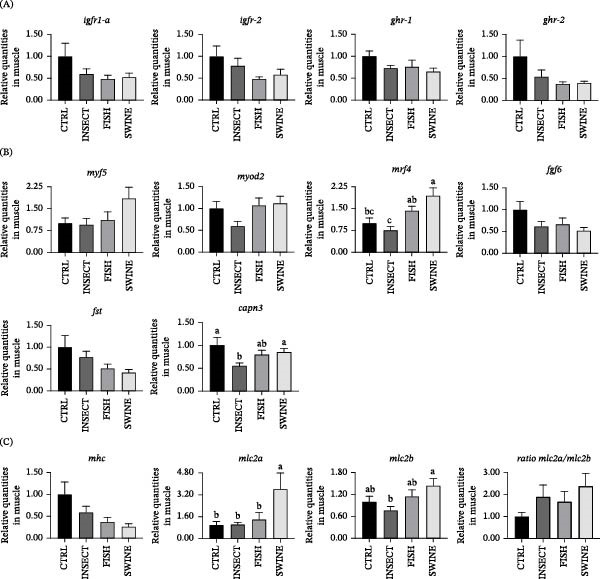
Gene expression in the muscle of gilthead seabream fed the experimental diets (CTRL, INSECT, FISH and SWINE) over 88 days. Relative gene expression was calculated using the comparative Ct (ΔΔCt) method, with values normalised to the CTRL diet. Values are presented as mean ± SEM (*n* = 9 fish per treatment). (A) Genes related to growth (GH/ IGF system). (B) Genes involved in myogenesis, muscle growth regulators and calpain proteins. (C) Genes associated with muscle structure. In each row, different lowercase letters indicate significant differences between treatments (*p* < 0.05).

A panel of 19 genes involved in fast‐twitch skeletal muscle growth was analysed, with amplification efficiencies ranging from 93% to 108% (Table S2). Overall, the expression of GH/ IGF system‐related genes was similar among dietary treatments (Figure 1 and Table S3). Fish from the INSECT group exhibited reduced expression levels of *mrf4* and *capn3*. Specifically, *mrf4* expression was significantly reduced in the INSECT group relative to the FISH and SWINE groups, while *capn3* was significantly lower compared to the CTRL and SWINE groups. In addition, *myod2* exhibited the lowest expression in the INSECT group and tended to be downregulated (*p* = 0.060). In contrast, the expression levels of *mrf4*, *mlc2a* and *mlc2b* were higher in the SWINE group. In detail, the expression of *mrf4* was significantly higher compared to that of the CTRL and INSECT groups. *mlc2a* was also significantly upregulated in SWINE relative to all other diets, while *mlc2b* differed significantly only from the INSECT group. Conversely, *fst* expression levels were lowest in the SWINE group, showing a tendency to be downregulated (*p* = 0.061). Gene expression levels were similar between fish fed the FISH and CTRL diets.

## 4. Discussion

PHs are well‐established as valuable functional ingredients in aquafeeds, with potential benefits for fish growth performance and robustness [8, 11]. In the present study, replacing 3% of CPSP90, a high‐value commercial PH, with alternative PHs, did not affect growth, nutrient utilisation, feed efficiency or anterior intestine integrity. However, fish fed the SWINE diet showed upregulation of key muscle growth‐related genes, including *mrf4* and *mlc2a*, suggesting stimulation of myogenic and muscle structural pathways that may support longer‐term growth. While most studies have investigated PHs as FM replacements [43–45], the present study directly compared diets with the same basal formulation, differing solely in the type of hydrolysate included.

The inclusion of alternative PHs at 3% did not significantly affect nutrient digestibility. Although PHs can exert physiological effects beyond their nutritional contribution through bioactive peptides and specific AAs that influence digestive processes and nutrient absorption [8, 11], ADCs remained similar among treatments. Previous studies have reported improvements at low dietary PH inclusion levels. For example, Zheng et al. [46, 47] reported that replacing FM with 1.2%–3.7% fish PHs significantly increased DM and protein ADCs in turbot (*Scophthalmus maximus* L.) and Japanese flounder (*Paralichthys olivaceus*), with the highest values obtained at 3.7%, a level comparable to the 3% inclusion used in the present study. In contrast, the inclusion of 1%–3% porcine hydrolysates did not affect nutrient digestibility in gilthead seabream or European seabass (*Dicentrarchus labrax*), with no changes in DM, protein or energy ADCs relative to FM‐based control diets [44, 48]. However, those studies assessed FM replacement, whereas the present work evaluated the replacement of CPSP90, a commercial PH previously reported to have high apparent AA digestibility in fish [49]. Overall, in the present study, ADC values were within the expected range for seabream [36, 50], and replacing CPSP90 with alternative PHs resulted in similar diet digestibility. Although ADCs were unaffected, faecal nutrient losses varied slightly among treatments, with higher energy losses in CTRL and lower lipid losses in INSECT. In addition, FISH and SWINE showed higher DPhos intake and faecal Phos losses than CTRL. However, Phos ADC and retention efficiency were unchanged, suggesting that these differences were mainly driven by Phos intake rather than altered Phos utilisation.

In the present study, histological analysis of the anterior intestine exhibited preserved mucosal integrity across all treatments, corroborating the digestibility results. The analysed morphometric parameters, such as absorption area, villi length and GC quantification, were similar among dietary treatments and values are consistent with those previously reported in the literature for gilthead seabream [51, 52]. GCs are mucin‐producing cells involved in intestinal protection, lubrication and nutrient absorption and their proliferation is often associated with increased mucus production [53, 54]. Although no significant differences in GC were observed among treatments, fish fed the alternative PHs diets showed a tendency towards lower GC numbers (*p* = 0.054). In pigs, a link between reduced GC quantity and improved feed efficiency was proposed, suggesting that lower mucus production may favour nutrient uptake or reduce the energetic cost of mucus synthesis, thereby improving feed utilisation [55]. However, in the present study, no significant differences in the FCR were observed.

Among treatments, fish fed the SWINE diet (containing the SH) exhibited numerically higher FBW, weight gain and DGI, although these were not statistically different from the remaining treatments. Gisbert et al. [45] reported that gilthead seabream juveniles, when fed a diet with a 5% inclusion of a swine PH (> 85% of peptides <10 kDa) at the expense of FM, exhibited higher FBW against a plant‐based control diet. The authors attributed this improvement to increased feed intake, likely driven by the enhanced palatability of the diet due to the inclusion of a swine PH. Although the SH used in the present study displayed a similar peptide profile (> 74% of peptides <10 kDa; Monteiro et al. [26]), no significant differences were observed in fish FBW or VFI.

Previous research has highlighted the beneficial role of low MW peptides (<1–2 kDa) in enhancing fish growth, feed efficiency and nutrient utilisation [43, 47, 56]. The four PHs included in this study, previously characterised by Monteiro et al. [26], differed in peptide size distribution and AA profiles. SH and CPSP90 displayed a broader peptide size distribution, with a lower proportion of low‐MW peptides (<1 kDa: 14%–26%) and low‐to‐medium‐MW (1–3 kDa: 28%–39%). In contrast, the IH and FH contained higher proportions of low‐MW (<1 kDa: 35%–36%) and low‐to‐medium‐MW (1–3 kDa: 51%–57%) peptides, a profile more consistent with that associated with improved growth performance in the literature [43, 47, 56]. Despite this, fish fed the SWINE diet exhibited the highest FBW, although the differences were not statistically significant. This could indicate that the peptide size distribution alone does not fully explain growth outcomes. Notably, SH was characterised by a higher NEAA contribution, which resulted in a higher proportion of NEAA in the SWINE diet, particularly collagenic AA, such as glycine and proline [57]. In line with this, comparable findings between increased NEAA availability and improved growth and feed utilisation were described in Nile tilapia (*Oreochromis niloticus*) [58]. Overall, these findings suggest that both peptide size distribution and AA composition contribute to the nutritional value of PHs and may modulate fish growth performance [11, 59].

To further explore the potential mechanisms underlying growth outcomes, muscle gene expression was analysed. Recent studies suggest that PHs can modulate the expression of myogenic regulatory factors (MRFs, e. g., *myod*, *myog*, *myf5* and *mrf4*), thereby influencing muscle growth dynamics in fish [12, 60]. For instance, in juvenile barramundi (*Lates calcarifer*) fed a diet in which poultry by‐product meal was the sole protein source, supplementation with 10% fish PH alleviated the negative effects on muscle growth and was accompanied by higher expression of MRFs, particularly *myog* and *myf5* [12]. Moreover, in juvenile European seabass fed a plant‐based diet, supplementation with 3% porcine blood PH was shown to modulate MRF expression, including a downregulation of *myod1*, which was associated with reduced fibre recruitment and muscle growth [60]. In the current study, fish fed the SWINE diet showed significant upregulation of *mrf4*, a key regulator of myogenesis associated with multinucleated myotube development and late‐stage differentiation [61, 62]. Additionally, *mlc2a* and *mlc2b*, components of the myosin complex involved in muscle contraction and markers of the early stages of myogenesis, were also upregulated. The upregulation of *mrf4*, together with increased expression of the structural genes *mlc2a* and *mlc2b*, suggests that late stages of myogenic differentiation and the expression of muscle structural components may have been stimulated [63, 64]. In contrast, *fst* (follistatin), a gene that encodes an activin‐binding protein involved in muscle fibre formation, and whose overexpression has been associated with increased muscle mass [65, 66], tended to be downregulated (*p* = 0.061) in the SWINE group. However, as growth performance did not differ significantly among treatments, these gene expression changes should be interpreted cautiously and cannot be directly associated with improved growth under the present experimental conditions. Nevertheless, muscle histological assessment combined with ultrastructural analysis of the muscle tissue could be a valuable tool to clarify whether these molecular responses are reflected at the phenotypic level [13]. In addition, longer‐term feeding trials under commercial production conditions would be useful to determine whether these responses translate into measurable effects on the growth performance.

The INSECT group also showed significant differences in skeletal muscle gene expression. Specifically, *myod2*, a member of the MRF family involved in myoblast determination, tended to be downregulated (*p* = 0.060). In line with this, Moutinho et al. [67] reported downregulation of *myod2* in the muscle of gilthead seabream fed diets containing *Hermetia illucens* insect meal, while *myod1* expression remained unaffected and no differences in growth performance were observed. Similarly, Basto et al. [68] described downregulation of *myod2* in the muscle of European seabass‐fed *Tenebrio molitor* insect meal, also without effects on growth. Although *myod2* downregulation may suggest altered regulation of muscle growth, overall growth was not affected in the present study and skeletal muscle cellularity was not assessed, preventing definitive conclusions. Moreover, *capn3*, a cysteine protease involved in muscle protein turnover and sarcomere integrity [69], was downregulated in the INSECT group. In mammals, altered *capn3* expression or function has been linked to increased adiposity and alterations in the lipid metabolism. For instance, the reduced expression of this gene in human skeletal muscle has been associated with obesity‐related phenotypes and insulin resistance [70, 71]. Moreover, in mice, *capn3* knock‐out models show altered expression of genes involved in lipid metabolism and impaired activation of the FOXO transcription factor, which contributes to lipid utilisation under stressful conditions [72, 73]. In the current study, the INSECT group showed significantly lower faecal lipid losses, suggesting differences in lipid utilisation. Elevated plasma triglyceride levels in the INSECT group (CTRL = 2.82 mmol L^−1^ and INSECT = 3.52 mmol L^−1^) [74] may further support altered lipid handling as triglycerides are major lipid transport and storage molecules in fish and a reliable indicator of lipid reserves [75]. However, HSI and VSI remained similar among groups. Moreover, the SWINE group also exhibited significantly lower faecal lipid losses, with lipid gain and WBC lipid levels comparable to those observed in the INSECT group, while the *capn3* expression remained unchanged. Therefore, a direct link between *capn3* expression and lipid utilisation cannot be established from the present data. Monteiro et al. [26] reported that IH exhibited high protein solubility (61%–65% between pH 3–7), substantially greater than that of other hydrolysates (26%–54%). Higher protein solubility of an ingredient has been associated with improved lipid dispersion and interaction during digestion, promoting micelle formation and facilitating lipid transport across the intestinal epithelium [76, 77]. These functional properties may have contributed to the lower faecal lipid losses observed in the INSECT group. Overall, the INSECT diet did not significantly affect the growth performance and the precise mechanisms linking PH inclusion, lipid metabolism and *capn3* expression remain to be elucidated.

## 5. Conclusion

The present study demonstrates the potential of alternative PHs as viable replacements for CPSP90 in practical aquafeed formulations. Replacing 3% of CPSP90 with alternative PHs resulted in similar nutrient utilisation, growth performance, feed efficiency and intestinal morphology under the present experimental conditions. Although growth performance did not differ among treatments, fish fed the SWINE diet showed upregulation of muscle growth gene expression (*mrf4*, *mlc2a* and *mlc2b*), suggesting modulation of myogenic differentiation and muscle structural pathways. Fish fed the INSECT diet displayed lower faecal lipid losses, accompanied by *capn3* downregulation, which may indicate metabolic adjustments related to lipid utilisation. The ability of alternative PHs to sustain growth performance, feed efficiency, nutrient utilisation and intestinal morphology at levels comparable to the control diet supports their use as CPSP90 replacements, contributing to the valorisation of locally sourced ABPs and insect meal within circular feed systems. Future studies should evaluate their long‐term effects on growth performance, muscle development and lipid metabolism.

## Acknowledgments

During the preparation of this article, ChatGPT (OpenAI, accessed during 2025−2026) was used to assist with minor language editing, translation of small text segments from Portuguese to English and improvements to text clarity and flow.

## Funding

This research received funding from the Blue Bioeconomy Pact (Grant C644915664‐00000026), specifically for work conducted under WP6 FEED as part of the Pep4Fish project, supported by international resources from the European Union. Additional support was provided by the Fundação para a Ciência e a Tecnologia (FCT, I.P.) through national funds and from the European Commission via the Recovery and Resilience Facility. Funding was provided within the scope of UIDB/04423/2025 (https://doi.org/10.54499/UIDB/04423/2025), UID/ PRR/04423/2025 (https://doi.org/10.54499/UID/PRR/04423/2025) and LA/ P/0101/2020 (https://doi.org/10.54499/LA/P/0101/2020). A. Alves‐de‐Oliveira acknowledges FCT for her PhD scholarship (2023.02611.BD, https://doi.org/10.54499/2023.02611.BD).

## Disclosure

All outputs were critically reviewed and edited by the authors, who take full responsibility for the integrity and accuracy of the manuscript.

## Conflicts of Interest

A. Almeida is affiliated with ETSA Group through employment. The other authors declare no conflicts of interest.

## Supporting Information

Additional supporting information can be found online in the Supporting Information section.

## Supporting information


**Supporting Information** Table S1. 1SFAs sum includes C10:0, C12:0, C13:0, C14:0, C15:0, C16:0, C17:0, C18:0, C20:0, C21:0, C22:0 and C24:0; 2MUFAs sum includes : C14:1, C16:1, C17:1 n7, C18:1 n9, C18:1 n7, C20:1 n9, C22:1 n11, C22:1 n9 and C24:1 n9; 3PUFAs sum includes: C16:2 n4, C16:3 n4, C16:4 n1, C18:2 n6, C18:3 n6, C18:3 n3, C18:4 n3, C20:2 n6, C20:3 n6, C20:3 n3, C20:4 n‐6, C20:4 n3, C20:5 n3, C22:2 n6, C22:5 n3 and C22:6 n3. AL, α‐linolenic acid; DHA, docosahexaenoic; DM, dry matter, EPA, eicosapentaenoic acid; LA, linoleic acid; MUFA, monounsaturated fatty acids; OA, oleic acid; PA, palmitoleic acid; PUFA, polyunsaturated fatty acids; SFA, saturated fatty acids. Table S2. Target and reference genes and the corresponding primers used in the reverse transcription qPCR analysis performed in the muscle tissues. For each primer pair, it is indicated the oligonucleotide sequence, annealing temperature (°C), the qPCR amplification efficiency (%), the GenBank accession number and the source reference for the primers. Table S3. Gene expression levels of *Sparus aurata* in muscle tissue after being fed the experimental diets for 88 days. Figure S1. Transverse cross‐sections of the anterior intestine (stained with AB/ PAS) of *Sparus aurata* fed the experimental diets. CSP, cross‐sectional perimeter; LP, lamina propria width; M, muscularis thickness; SM, submucosa thickness; VL, villi length.

## Data Availability

Access to the datasets produced during this work may be granted by the corresponding author upon request.
