# Supplementary material for: Upcycled Animal Protein Hydrolysates for Gilthead Seabream: Impact on Growth Performance, Nutrient Utilisation, Intestinal Structure and Skeletal Muscle Gene Expression
Source: Aquac Nutr. 2026 Jul 17;2026:2611411. doi: 10.1155/anu/2611411 (PMC13379556; doi:10.1155/anu/2611411)
Supplement: Supplementary file 1 — Supporting Information Table S1. 1SFAs sum includes C10:0, C12:0, C13:0, C14:0, C15:0, C16:0, C17:0, C18:0, C20:0, C21:0, C22:0 and C24:0; 2MUFAs sum includes : C14:1, C16:1, C17:1 n7, C18:1 n9, C18:1 n7, C20:1 n9, C22:1 n11, C22:1 n9 and C24:1 n9; 3PUFAs sum includes: C16:2 n4, C16:3 n4, C16:4 n1, C18:2 n6, C18:3 n6, C18:3 n3, C18:4 n3, C20:2 n6, C20:3 n6, C20:3 n3, C20:4 n‐6, C20:4 n3, C20:5 n3, C22:2 n6, C22:5 n3 and C22:6 n3. AL, α‐linolenic acid; DHA, docosahexaenoic; DM, dry matter, EPA, eicosapentaenoic acid; LA, linoleic acid; MUFA, monounsaturated fatty acids; OA, oleic acid; PA, palmitoleic acid; PUFA, polyunsaturated fatty acids; SFA, saturated fatty acids. Table S2. Target and reference genes and the corresponding primers used in the reverse transcription qPCR analysis performed in the muscle tissues. For each primer pair, it is indicated the oligonucleotide sequence, annealing temperature (°C), the qPCR amplification efficiency (%), the GenBank accession number and the source reference for the primers. Table S3. Gene expression levels of Sparus aurata in muscle tissue after being fed the experimental diets for 88 days. Figure S1. Transverse cross‐sections of the anterior intestine (stained with AB/ PAS) of Sparus aurata fed the experimental diets. CSP, cross‐sectional perimeter; LP, lamina propria width; M, muscularis thickness; SM, submucosa thickness; VL, villi length. [file ANU-2026-2611411-s001.docx]

| **Table S1.** Fatty acid profile of the experimental diets. | | | | |
| --- | --- | --- | --- | --- |
|  | **CTRL** | **INSECT** | **FISH** | **SWINE** |
| **Fatty acids (g 100 g^-1^ DM)** | | | | |
| C14:0 | 0.43 | 0.46 | 0.48 | 0.48 |
| C16:0 | 2.25 | 2.41 | 2.48 | 2.53 |
| C18:0 | 0.59 | 0.63 | 0.65 | 0.67 |
| Σ SFA^1^ | 3.60 | 3.81 | 3.90 | 3.97 |
| C16:1 n7 (PA) | 0.65 | 0.70 | 0.72 | 0.73 |
| C18:1 n70.343 | 0.49 | 0.52 | 0.53 | 0.54 |
| C18:1 n9 (OA) | 6.11 | 6.43 | 6.59 | 6.66 |
| C20:1 n9 | 0.16 | 0.15 | 0.15 | 0.15 |
| ΣMUFA^2^ | 7.52 | 7.89 | 8.09 | 8.19 |
| C18:2 n6 (LA) | 2.22 | 2.26 | 2.32 | 2.33 |
| C18:3 n3 (ALA) | 0.53 | 0.55 | 0.56 | 0.56 |
| C18:4 n3 | 0.08 | 0.09 | 0.09 | 0.09 |
| C20:4 n6 | 0.09 | 0.10 | 0.11 | 0.11 |
| C20:4 n3 | 0.04 | 0.04 | 0.04 | 0.04 |
| C20:5 n3 (EPA) | 1.09 | 1.15 | 1.18 | 1.19 |
| C22:5 n3 | 0.08 | 0.14 | 0.14 | 0.15 |
| C22:6 n3 (DHA) | 0.47 | 0.50 | 0.51 | 0.53 |
| EPA + DHA | 1.56 | 1.65 | 1.69 | 1.72 |
| ΣPUFA^3^ | 4.66 | 4.88 | 5.01 | 5.06 |
| ΣPUFA n3 | 2.30 | 2.47 | 2.53 | 2.56 |
| ΣPUFA n6 | 2.36 | 2.41 | 2.48 | 2.50 |
| ΣPUFA n3/ΣPUFA n6 | 0.97 | 1.02 | 1.02 | 1.03 |
| ^1^SFAs sum includes C10:0, C12:0, C13:0, C14:0, C15:0, C16:0, C17:0, C18:0, C20:0, C21:0, C22:0 and C24:0; ^2^MUFAs sum includes : C14:1, C16:1, C17:1 n7, C18:1 n9, C18:1 n7, C20:1 n9, C22:1 n11, C22:1 n9 and C24:1 n9; ^3^PUFAs sum includes: C16:2 n4, C16:3 n4, C16:4 n1, C18:2 n6, C18:3 n6, C18:3 n3, C18:4 n3, C20:2 n6, C20:3 n6, C20:3 n3, C20:4 n-6, C20:4 n3, C20:5 n3, C22:2 n6, C22:5 n3 and C22:6 n3 . The abbreviations stand for: DM, dry matter, ALA. α-linolenic acid; DHA, docosahexaenoic; EPA, eicosapentaenoic acid; LA, linoleic acid; MUFA, monounsaturated fatty acids; OA, oleic acid; PA, palmitoleic acid; PUFA, polyunsaturated fatty acids; SFA, saturated fatty acids. | | | | |

| **Table S2.** Target and reference genes and the corresponding primers used in the reverse transcription qPCR analysis performed in the muscle tissues. For each primer pair, it is indicated the oligonucleotide sequence, annealing temperature (°C), the qPCR amplification efficiency (%), the GenBank accession number and the source reference for the primers. | | | | | | |
| --- | --- | --- | --- | --- | --- | --- |
| **Target** | **Primer sequence Fw (5′-3′)** | **Primer sequence Rv (5′-3′)** | **Ta (°C)** | **Amplification efficiency (%)** | **Accession number** | **Reference** |
| **Reference genes** | | | | | | |
| *rps18* | TGACGGAAGGGCACCACCAG | AATCGCTCCACCAACTAAGAACGG | 60 | 107 | [AY550956](https://www.ncbi.nlm.nih.gov/nuccore/AY550956) | Perelló-Amorós [1] |
| *rpl27α* | AAGAGGAACACAACTCACTGCCCCAC | GCTTGCCTTTGCCCAGAACTTTGTAG | 60 | 96 | [AY188520](https://www.ncbi.nlm.nih.gov/nuccore/AY188520) | Perelló-Amorós [1] |
| ***GH/IGF* system** | | | | | | |
| *igfr-1a* | TCAACGACAAGTACGACTACCGCTGCT | CACACTTTCTGGCACTGGTTGGAGGTC | 60 | 106 | [KJ591052](https://www.ncbi.nlm.nih.gov/nuccore/KJ591052) | Ramos-Pinto [2] |
| *igfr-2* | ACCTGTCAGCCACCACATGA | TCGTGCAGATCTGGGTCGTA | 60 | 101 | [KM522776](https://www.ncbi.nlm.nih.gov/nuccore/KM522776) | Ramos-Pinto [2] |
| *ghr-1* | ACCTGTCAGCCACCACATGA | TCGTGCAGATCTGGGTCGTA | 60 | 107 | [AF438176](https://www.ncbi.nlm.nih.gov/nuccore/AF438176) | Otero-Tarrazón [3] |
| *ghr-2* | GAGTGAACCCGGCCTGACAG | GCGGTGGTATCTGATTCATGGT | 60 | 96 | [AY573601](https://www.ncbi.nlm.nih.gov/nuccore/AY573601) | Otero-Tarrazón [3] |
| **Genes involved in myogenesis** | | | | | | |
| *myf5* | GCATGGTTGACAGCAACAGTCCAGTGT | TGTCTTATCGCCCAAAGTGTCGTTCTTCAT | 60 | 97 | [JN034420](https://www.ncbi.nlm.nih.gov/nuccore/JN034420) | Calduch-Giner [4] |
| *myod1* | GTTTTGTTCCAGGCGGTCT | GCTGGTGTCGGTGGAGAT | 60 | 100 | [AF478568](https://www.ncbi.nlm.nih.gov/nuccore/AF478568) | Ramos-Pinto [2] |
| *myod2* | CACTACAGCGGGGATTCAGAC | CGTTTGCTTCTCCTGGACTC | 55 | 101 | [AF478569](https://www.ncbi.nlm.nih.gov/nuccore/AF478569) | Ramos-Pinto [2] |
| *myog* | CAGAGGCTGCCCAAGGTGGAG | CAGGTCCTGCCCGAACTGGGCTCG | 68 | 108 | [EF462191](https://www.ncbi.nlm.nih.gov/nuccore/EF462191) | Jiménez-Amilburu [5] |
| *mrf4* | CATCCCACAGCTTTAAAGGCA | GAGGACGCCGAAGATTCACT | 60 | 98 | [JN034421](https://www.ncbi.nlm.nih.gov/nuccore/JN034421) | Perelló-Amorós [1] |
| *mymk* | TTCACTGCGGTTTACCACGC | CCCACATAGAGAGAGCTGTGCTG | 55 | 95 | [XM_030418477.1](https://www.ncbi.nlm.nih.gov/nuccore/XM_030418477.1) | Perelló-Amorós [1] |
| *fgf6* | TTGCTCATATTCGGGGGTAG | AGCTTGCTCCCGAACAACTA | 55 | 99 | [XM_030425434.1](https://www.ncbi.nlm.nih.gov/nucleotide/XM_030425434.1?report=genbank&log$=nucltop&blast_rank=1&RID=64YR48CE016) | Santos [6] |
| **Muscle growth regulators** | | | | | | |
| *mstn* | GTACGACGTGCTGGGAGACG | CGTACGATTCGATTCGCTTG | 57 | 93 | [AF258448.1](https://www.ncbi.nlm.nih.gov/nuccore/AF258448.1) | Ramos-Pinto [2] |
| *fst* | GGACCAGACAAACAACGCATATTG | CATAGATGATCCCGTCGTTTCCAC | 60 | 102 | [AY544167](https://www.ncbi.nlm.nih.gov/nuccore/AY544167) | Calduch-Giner [4] |
| *murf1* | GTGACGGCGAGGATGTGC | CTTCGGCTCCTTGGTGTCTT | 60 | 93 | [FM145056](https://www.ncbi.nlm.nih.gov/nuccore/FM145056) | Otero-Tarrazón [3] |
| **Calpain proteins** | | | | | | |
| *capn1* | CCTACGAGATGAGGATGGCT | AGTTGTCAAAGTCGGCGGT | 60 | 96 | [AM951595.1](https://www.ncbi.nlm.nih.gov/nuccore/AM951595.1) | Ramos-Pinto [2] |
| *capn3* | AGAGGGTTTCAGCCTTGAGA | CGCTTTGATCTTTCTCCACA | 55 | 106 | [FG262721.1](https://www.ncbi.nlm.nih.gov/nuccore/FG262721.1) | Ramos-Pinto [2] |
| **Muscle structural genes** | | | | | | |
| *mhc* | AGCAGATCAAGAGGAACAGCC | GACTCAGAAGCCTGGCGATT | 60 | 107 | [NM131404](https://www.ncbi.nlm.nih.gov/nuccore/NM_131404.2/) | Ramos-Pinto [2] |
| *mlc2a* | GCCCCATCAACTTCACCGTCTTT | GGTTGGTCATCTCCTCAGCGG | 60 | 102 | [AF150904](https://www.ncbi.nlm.nih.gov/nuccore/AF150904) | Georgiou [7] |
| *mlc2b* | TCCCTTTGCTATTCTGCCTTC | AAATCAGCCCTATTCCCCATA | 55 | 103 | [FG618629](https://www.ncbi.nlm.nih.gov/nuccore/FG618629) | Georgiou [7] |
| The abbreviations stand for: Ta, annealing temperature; *rps18*, ribosomal protein s18; *rpl27a*, ribosomal protein l27a; *igfr-1a*, insulin-like growth factor I receptor a; *igfr-2*, cation-independent mannose-6-phosphate receptor; *ghr-1,* growth hormone receptor 1; *ghr-2,* growth hormone receptor 2; *myf5*, myogenic factor-like 5; *myod1*, myoblast determination protein 1; *myod2*, myoblast determination protein 2; *myog,* myogenin; *mrf4,* myogenic regulatory factor 4; *mymk*, myomaker; *fgf6*, fibroblast growth factor 6; *mstn*, myostatin; *fst,* follistatin; *murf1,* muscle RING finger protein-1; *capn1*, calpain 1; *capn3*, calpain 3; *mhc*, myosin heavy chain; *mlc2a*, myosin light chain 2a isoform A; *mlc2b*, myosin light chain 2a isoform B. | | | | | | |

| **Table S3.**  Gene expression levels of *Sparus aurata* in muscle tissue after being fed the experimental diets for 88 days. | | | | | |
| --- | --- | --- | --- | --- | --- |
| **Gene** | **CTRL** | **INSECT** | **FISH** | **SWINE** | **p-value** |
| *igfr-1a* | 1.000 ± 0.297 | 0.597 ± 0.115 | 0.486 ± 0.084 | 0.525 ± 0.092 | 0.194 |
| *igfr-2* | 1.000 ± 0.234 | 0.783 ± 0.172 | 0.482 ± 0.047 | 0.580 ± 0.124 | 0.116 |
| *ghr-1* | 1.000 ± 0.119 | 0.730 ± 0.062 | 0.761 ± 0.149 | 0.653 ± 0.076 | 0.120 |
| *ghr-2* | 1.000 ± 0.372 | 0.540 ± 0.150 | 0.374 ± 0.045 | 0.397 ± 0.036 | 0.623 |
| *myf5* | 1.000 ± 0.178 | 0.952 ± 0.215 | 1.116 ± 0.274 | 1.852 ± 0.382 | 0.090 |
| *myod1* | 1.000 ± 0.162 | 0.930 ± 0.126 | 0.894 ± 0.118 | 0.842 ± 0.147 | 0.877 |
| *myod2* | 1.000 ± 0.156 | 0.596 ± 0.110 | 1.071 ± 0.171 | 1.119 ± 0.161 | 0.060 |
| *myog* | 1.000 ± 0.467 | 0.544 ± 0.125 | 0.543 ± 0.138 | 0.878 ± 0.207 | 0.671 |
| *mrf4* | 1.000 ± 0.175^bc^ | 0.757 ± 0.124^c^ | 1.432 ± 0.148^ab^ | 1.945 ± 0.267^a^ | <0.001 |
| *mymk* | 1.000 ± 0.210 | 0.994 ± 0.279 | 1.083 ± 0.440 | 1.792 ± 0.818 | 0.860 |
| *fgf6* | 1.000 ± 0.186 | 0.617 ± 0.115 | 0.665 ± 0.144 | 0.521 ± 0.072 | 0.094 |
| *mstn* | 1.000 ± 0.300 | 2.568 ± 0.881 | 2.686 ± 0.928 | 1.818 ± 0.813 | 0.418 |
| *fst* | 1.000 ± 0.264 | 0.773 ± 0.134 | 0.514 ± 0.098 | 0.422 ± 0.067 | 0.061 |
| *murf1* | 1.000 ± 0.305 | 0.682 ± 0.137 | 0.458 ± 0.049 | 0.558 ± 0.083 | 0.750 |
| *capn1* | 1.000 ± 0.187 | 0.731 ± 0.067 | 0.737 ± 0.092 | 0.978 ± 0.144 | 0.352 |
| *capn3* | 1.000 ± 0.180^a^ | 0.552 ± 0.065^b^ | 0.797 ± 0.098^ab^ | 0.853 ± 0.076^a^ | 0.047 |
| *mhc* | 1.000 ± 0.280 | 0.589 ± 0.139 | 0.370 ± 0.103 | 0.262 ± 0.068 | 0.148 |
| *mlc2a* | 1.000 ± 0.256^b^ | 1.043 ± 0.152^b^ | 1.401 ± 0.498^b^ | 3.615 ± 1.178^a^ | 0.009 |
| *mlc2b* | 1.000 ± 0.149^ab^ | 0.763 ± 0.107^b^ | 1.145 ± 0.179^ab^ | 1.444 ± 0.190^a^ | 0.049 |
| ratio *mlc2a/mlc2b* | 1.000 ± 0.191 | 1.895 ± 0.543 | 1.674 ± 0.472 | 2.384 ± 0.587 | 0.133 |
| Values are the mean ± SEM (n = 9 per treatment). Relative gene expression was calculated using the 2^−ΔΔCT^ method, with values normalised to the CTRL diet. In each row, different letters indicate significant differences between treatments (p < 0.05). | | | | | |


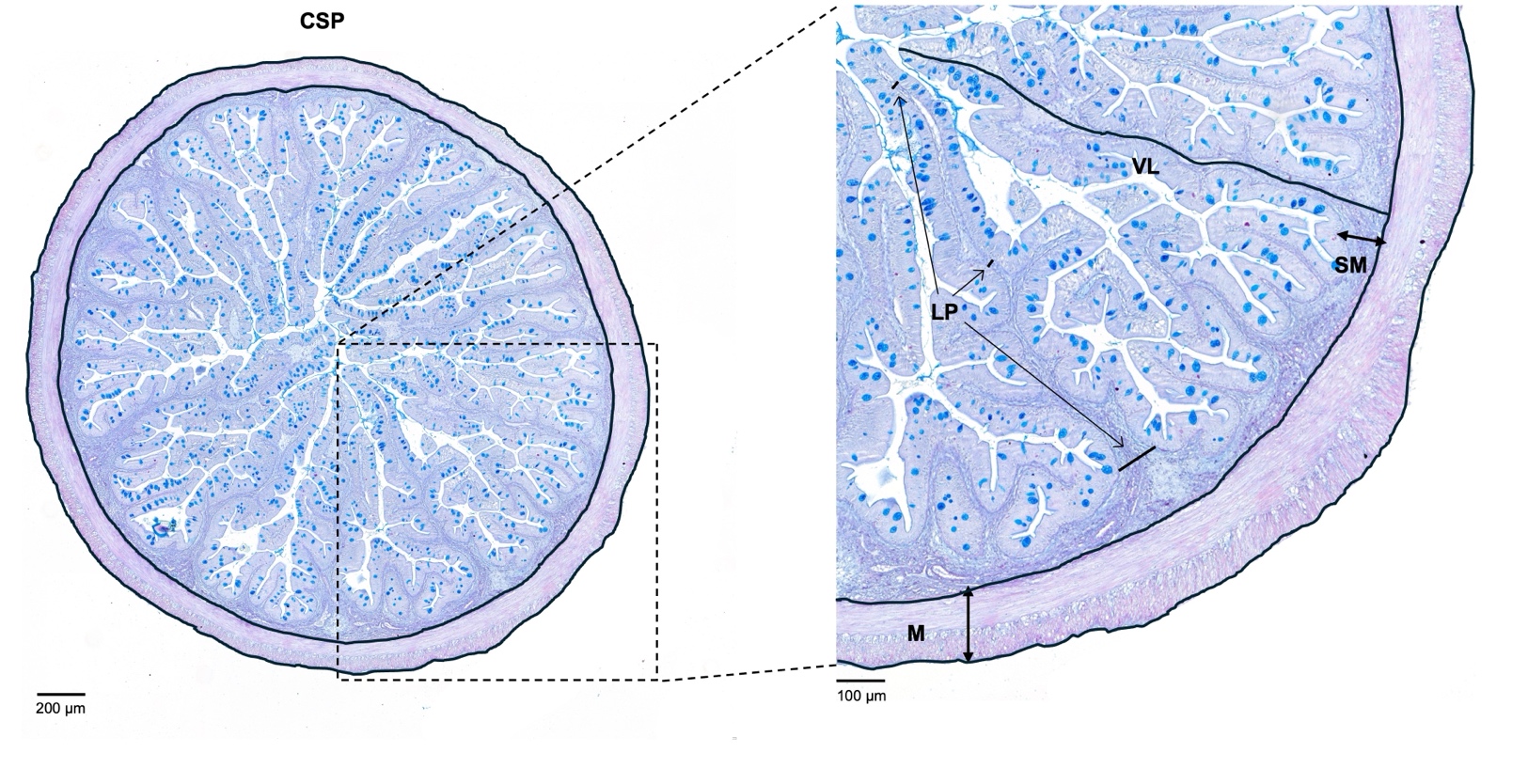
**Figure S1.** Transverse cross-sections of the anterior intestine (stained with AB/PAS) of *Sparus aurata* fed the experimental diets. The abbreviations stand for: CSP, cross-sectional perimeter; M, *muscularis* thickness; SM, submucosa thickness; VL, *villi* length; LP, lamina propria width.

References

1. Perelló-Amorós M., Otero-Tarrazón A., Jorge-Pedraza V., García-Pérez I., Sánchez-Moya A., Gabillard J.-C., Moshayedi F., Navarro I., Capilla E., Fernández-Borràs J., Blasco J., Chillarón J., García De La Serrana D., Gutiérrez J. Myomaker and Myomixer Characterization in Gilthead Sea Bream under Different Myogenesis Conditions. *International Journal of Molecular Sciences*. (2022) 23, no. 23, 14639. <https://doi.org/10.3390/ijms232314639>.

2. Ramos-Pinto L., Lopes G., Sousa V., Castro L. F. C., Schrama D., Rodrigues P., Valente L. M. P. Dietary Creatine Supplementation in Gilthead Seabream (*Sparus aurata*) Increases Dorsal Muscle Area and the Expression of myod1 and capn1 Genes. *Frontiers in Endocrinology*. (2019) 10, 1-13. <https://doi.org/10.3389/fendo.2019.00161>.

3. Otero-Tarrazón A., Perelló-Amorós M., Jorge-Pedraza V., Moshayedi F., Sánchez-Moya A., García-Pérez I., Fernández-Borràs J., García De La Serrana D., Navarro I., Blasco J., Capilla E., Gutiérrez J. Muscle regeneration in gilthead sea bream: Implications of endocrine and local regulatory factors and the crosstalk with bone. *Frontiers in Endocrinology*. (2023) 14, 1-13. <https://doi.org/10.3389/fendo.2023.1101356>.

4. Calduch-Giner J., Rosell-Moll E., Besson M., Vergnet A., Bruant J.-S., Clota F., Holhorea P. G., Allal F., Vandeputte M., Pérez-Sánchez J. Changes in transcriptomic and behavioural traits in activity and ventilation rates associated with divergent individual feed efficiency in gilthead sea bream (*Sparus aurata*). *Aquaculture Reports*. (2023) 29, 101476. <https://doi.org/10.1016/j.aqrep.2023.101476>.

5. Jiménez-Amilburu V., Salmerón C., Codina M., Navarro I., Capilla E., Gutiérrez J. Insulin-like growth factors effects on the expression of myogenic regulatory factors in gilthead sea bream muscle cells. *General and Comparative Endocrinology*. (2013) 188, 151-158. <https://doi.org/10.1016/j.ygcen.2013.02.033>.

6. Santos S., Tsipourlianos A., Angelakopoulos R., Li L., Power D. M., Moutou K. A. List of validated primers of gilthead sea bream (*Sparus aurata*) and European seabass (*Dicentrarchus labrax*) developed in PerformFISH project (D2.3) [Data set]. (2022). <https://doi.org/10.5281/zenodo.7271303>.

7. Georgiou S., Alami-Durante H., Power D. M., Sarropoulou E., Mamuris Z., Moutou K. A. Transient up- and down-regulation of expression of myosin light chain 2 and myostatin mRNA mark the changes from stratified hyperplasia to muscle fiber hypertrophy in larvae of gilthead sea bream (*Sparus* *aurata* L.). *Cell and Tissue Research*. (2016) 363, no. 2, 541-554. <https://doi.org/10.1007/s00441-015-2254-0>.
